# Gastroduodenal Polyposis Secondary to Extrahepatic Portal Venous Obstruction

**DOI:** 10.1002/jgh3.70060

**Published:** 2024-11-25

**Authors:** Christopher Wen Wei Ho, Kenneth Tou En Chang, Fang Kuan Chiou

**Affiliations:** ^1^ Gastroenterology, Hepatology and Nutrition Service, Paediatric Medicine KK Women's and Children's Hospital Singapore Singapore; ^2^ Department of Pathology and Laboratory Medicine KK Women's and Children's Hospital Singapore Singapore

**Keywords:** children, gastrointestinal bleed, polyp, portal hypertension

1

This is a 16‐year‐old male who first presented in infancy for poor weight gain and splenomegaly. Ultrasound and computed tomography imaging of the abdomen revealed extrahepatic portal vein obstruction (EHPVO) and portal hypertension, with chronic portal vein thrombosis, cavernous transformation of the portal vein, splenomegaly, and portal venous shunts.

He developed his first variceal bleed at 3 years old, with endoscopic variceal ligation of esophageal varices and injection sclerotherapy of gastric varices done successfully. Over the years, there was no recurrence of variceal bleed, though his spleen size had gradually increased in size with hypersplenic effect of leukopaenia, thrombocytopaenia, and anemia. There was no evidence of liver cirrhosis. At 16 years of age, he presented with hepatic encephalopathy and a drop in hemoglobin (from 10.4 to 7.0 g/DL) with suspected occult gastrointestinal bleeding. There was no overt haematemesis or melaena. Oesophagogastroduodenoscopy showed non‐bleeding Grade II esophageal varices for which endoscopic variceal ligation was performed. Multiple sessile polyps measuring approximately up to 5 mm were seen in the stomach antrum as well as in the second part of duodenum (Figure 1a–d). Overlying mucosa of these polyps appeared congested and although there was increased venous bleeding during biopsy, bleeding resolved without further intervention. Histology showed increased ectatic lamina proprial capillaries in the laminal propria with no dysplasia, findings which were in keeping with microscopic changes attributable to portal hypertension (Figure 1e).

**FIGURE 1:**
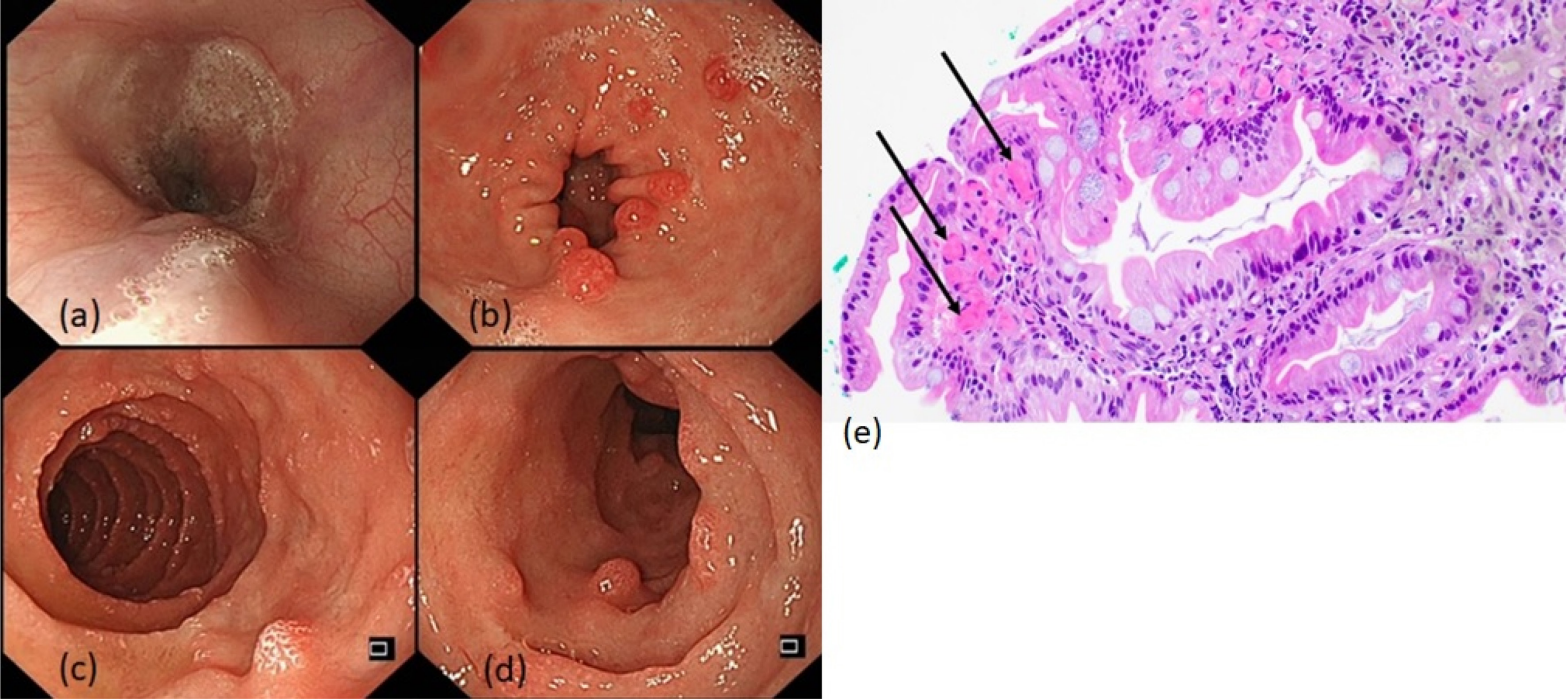
Endoscopic images showing (a) Grade II esophageal varix and multiple discrete sessile polyps scattered throughout in (b) the antrum of stomach and (c and d) second part of the duodenum, (e) Hematoxylin and eosin‐stained photomicrograph (magnification ×200) showing dilated and congested capillaries (black arrows) within the lamina propria of the duodenal mucosa.

Portal hypertensive polyps (PHP) have been described as a rare endoscopic feature of portal hypertension, along with other more common findings of oesphageal varices, gastropathy, gastric antral vascular ectasia, enteropathy, and colopathy [1]. It has been postulated that polyps develop because of neovascularization secondary to high portal pressure. PHP have been mainly described in the stomach and duodenal involvement is not common, with paucity of literature in children [2]. Differential diagnoses of PHP include pancreatic or gastric heterotopia, adenomatous polyps, and inflammatory polyps. Histological findings of proliferating capillaries in the lamina propria indicates a vascular etiology, distinguishing them from inflammatory polyps [3]. The absence of dysplasia rules out an adenomatous nature for these polyps. Other histological findings of PHP described are vascular ectasia/congestion/thrombi, gastric foveolar metaplasia, reactive nuclear atypia, fibrosis, and smooth muscle proliferation.

PHP have been associated with increased risk of bleeding due to underlying vascular congestion. In this case, the patient did not present with overt variceal bleeding, and ectopic bleeding from the PHP was postulated to have contributed to the anemia and triggered hepatic encephalopathy. Lowering portal pressure with beta‐blockers has shown reported improvement in both clinical (anemia and need for transfusions) and endoscopic features [1]. Small bowel mucosal changes may improve after transjugular intrahepatic portosystemic shunt (TIPS), but effect on PHP has not been reported. For asymptomatic patients, treatment may not be necessary except for endoscopic polypectomy for diagnostic purposes. This case highlights the importance of endoscopic and histologic evaluation of this unusual manifestation of portal hypertension as a source of occult gastrointestinal bleeding in patients with long‐standing portal hypertension.

## Ethics Statement

Approval by Institutional Review board was not required as this was a retrospective review of a patient's clinical results.

## Consent

Written consent has been obtained from the patient for publication.

## Conflicts of Interest

The authors declare no conflicts of interest.
